# Non-thermal air plasma promotes the healing of acute skin wounds in rats

**DOI:** 10.1038/srep45183

**Published:** 2017-03-24

**Authors:** S. Kubinova, K. Zaviskova, L. Uherkova, V. Zablotskii, O. Churpita, O. Lunov, A. Dejneka

**Affiliations:** 1 Institute of Experimental Medicine, Academy of Sciences of the Czech Republic, Prague, Czech Republic; 2 Institute of Physics, Academy of Sciences of the Czech Republic, Prague, Czech Republic; 3 2^nd^ Medical Faculty, Charles University, Prague, Czech Republic

## Abstract

Non-thermal plasma (NTP) has nonspecific antibacterial effects, and can be applied as an effective tool for the treatment of chronic wounds and other skin pathologies. In this study we analysed the effect of NTP on the healing of the full-thickness acute skin wound model in rats. We utilised a single jet NTP system generating atmospheric pressure air plasma, with ion volume density 5 · 10^17^ m^−3^ and gas temperature 30–35 °C. The skin wounds were exposed to three daily plasma treatments for 1 or 2 minutes and were evaluated 3, 7 and 14 days after the wounding by histological and gene expression analysis. NTP treatment significantly enhanced epithelization and wound contraction on day 7 when compared to the untreated wounds. Macrophage infiltration into the wound area was not affected by the NTP treatment. Gene expression analysis did not indicate an increased inflammatory reaction or a disruption of the wound healing process; transient enhancement of inflammatory marker upregulation was found after NTP treatment on day 7. In summary, NTP treatment had improved the healing efficacy of acute skin wounds without noticeable side effects and concomitant activation of pro-inflammatory signalling. The obtained results highlight the favourability of plasma applications for wound therapy in clinics.

Non-thermal plasma technology and its use in medicine (“plasma medicine”) has become a rapidly developing interdisciplinary field that brings a new innovative approach in a wide range of biomedical applications. Primarily due to its bactericidal properties, non-thermal plasma (NTP) represents an effective tool for various procedures in human as well as in veterinary medicine, particularly in tissue disinfection and treatment of chronic wounds, such as diabetic foot ulcers, pressure and venous leg ulcers, burns and other skin pathologies with microbial etiology^1^,^2^. Moreover, NTPs have shown their promising application also in cancer therapy^3^,^4^.

NTPs are generated from a flow of neutral gas in a locally high-strength electric field, while the gas remains at atmospheric pressure and near ambient temperature. Generally, NTP composition is very complex and includes excited particles, such as electrons, ions, reactive oxygen species (ROS, e.g. ozone – O_3_), reactive nitrogen species (RNS)^5^, and UV radiation^6^,^7^.

The bactericidal effects of NTP on bacteria can be explained by the deleterious impact of ionized particles on bacterial membranes, while the probable mechanisms could include membrane damage, membrane perforation by etching due to highly reactive gas radicals, or interactions with the negative and positive ions of the plasma, hydrogen peroxide, etc.^8^. We have previously demonstrated, that under plasma treatment, mechanically rigid bacterial wall structures can be destroyed due to internal electrostatic pressure raised, as a result of ion accumulation^9^. Depending on the plasma dose and voltage value producing the plasma discharges, NTP may trigger either programmed cell death or physical destruction of the bacteria^10^.

Generally, the accumulation of ROS/RNS species has been implicated to explain the underlying biological effects of non-thermal plasma^11^,^12^,^13^. Aside from bactericidal effects, ROS and other species generated by NTP may have favourable healing effects at the wound site, where they can directly function as signalling molecules. It is known that ROS play roles not only in disinfection during the inflammatory phase, but also in other phases involved in the regulation of tissue repair including migration, proliferation, and angiogenesis^14^. On the other hand, excessive ROS production may impede the healing process by creating an imbalanced redox homeostasis^15^.

Indeed, a positive effect of NTPs on wound healing without adverse reactions to the surrounding healthy tissue has been reported in animal studies^16^,^17^,^18^,^19^. Furthermore, randomised clinical trials have proven that NTPs can reduce bacteria load as well as promote the healing of chronic wounds, while no side-effects and good treatment tolerability were reported^20^,^21^,^22^,^23^.

However, despite the fact that distinct NTP devices have already been approved as safe in several clinical trials^10^,^11^,^12^,^13^, there are still many open issues with regard to the molecular or biophysical mechanisms of the biological effects of NTP on mammalian cells and tissue, as well as its potential role in the wound healing scenario. Therefore studies revealing the molecular mechanisms of plasma-cell interaction *in vivo* are indispensable for developing better and safe NTP therapies.

Remarkably, the biological effects of NTPs have been shown to be dose-dependent, ranging from stimulation of cell proliferation and migration^11^,^24^ to cell death by necrosis^25^ or apoptosis^12^,^26^. In this respect, using different plasma sources, operation parameters and other factors (working gas, plasma density, temperature, electric fields, ozone, UV, etc.) results in a substantial inconsistency of NTP effects on mammalian cells between various studies.

We have previously developed and characterized NTP system with controlled plasma composition and working temperature that has been shown to be an effective tool for bacterial eradication^27^,^28^. In our recent study we demonstrated that chemically distinct plasmas trigger different responses in mammalian cells, and that the extent of biological responses to NTP may grossly differ between phenotypically distinct cell lines^27^.

In this study, we investigated the safety and efficacy of air NTP treatment in skin wound healing, to verify the potential of our NTP system for future clinical application. As an experimental model, we used a full thickness skin wound in rats, which we evaluated by histological and gene expression analysis. We demonstrated that 1 min plasma exposure was efficient to kill Gramm-positive, as well as Gramm-negative bacteria *in vitro*. To reveal a potential dose dependent plasma effect on wound healing, we used 1 and 2 min plasma exposure.

## Results

### Wound closure

**Figure 1:**
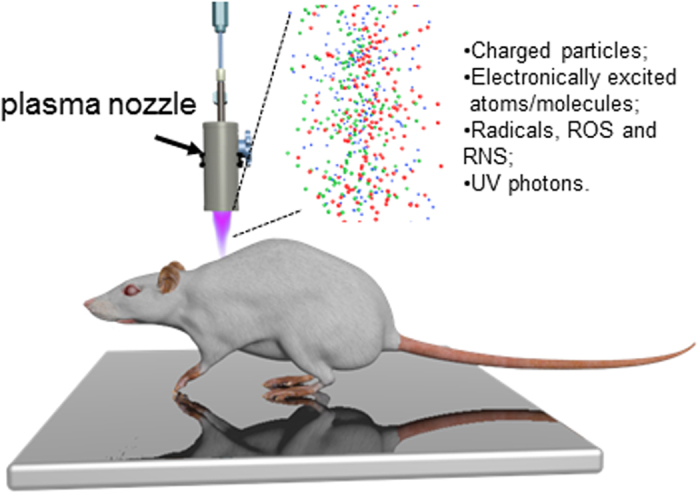
Schematic representation of the experimental procedure.

**Figure 2:**
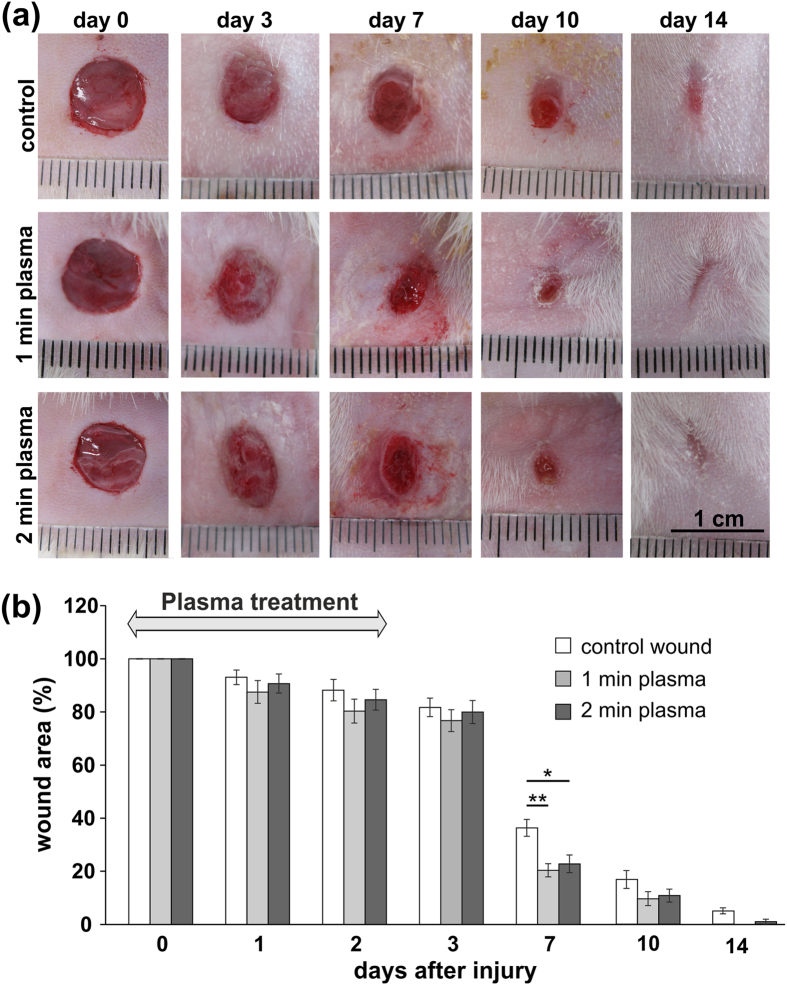
(**a**) Representative photographs of the full thickness skin wounds and subsequent wound contraction on days 3, 7, 10 and 14 in control and plasma treated wounds. Scale bar: 1 cm. (**b**) Quantification of the wound area in control and plasma treated wounds. Mean ± SEM, *p < 0.005, **p < 0.001.

The effects of NTP on wound healing were studied using a full-thickness skin wound model in rats. The general scheme of NTP rat treatment is shown in Fig. 1. The wound closure for each wound was evaluated on days 0–3, 7, 10 and 14 by determining the unclosed wound area in wounds treated with NTP for 1 or 2 minutes; the untreated wounds served as a control (Fig. 2a). Although no difference in wound closure was found among the groups in the early phase of the healing process (day 1–3), both 1 and 2 min of plasma treatment significantly improved wound closure on day 7 (p < 0.001, p < 0.005), compared with that in the control (Fig. 2b). The wound sizes of all groups at a later time interval on day 10 were not different, and the wounds were completely closed 14 days after the wounding. These data suggest wound healing potential of plasma treatment in the remodelling phase of the healing, while no significant differences in wound closure were observed between the wounds treated with 1 and 2 minutes of plasma exposure. Of note, no side effect of the NTP treatment, e.g. inflammation or erythema, were observed during the experiment.

### Histological analysis

**Figure 3:**
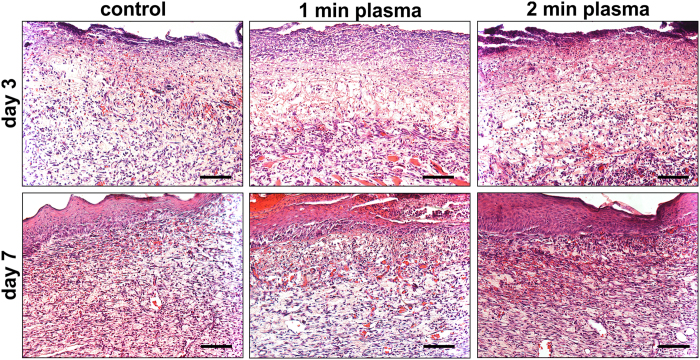
Representative histology of Hematoxylin-eosin staining of control and plasma treated wounds on days 3 and 7. Scale bars: 100 μm.

H&E staining (Fig. 3) was carried out on days 3 and 7, and did not show any notable differences between the control and plasma treated skin samples. On day 3, an infiltration of inflammatory cells was found in the control as well as plasma treated wounds. On day 7, all groups revealed a formation of blood vessels and wound epithelisation.

**Figure 4:**
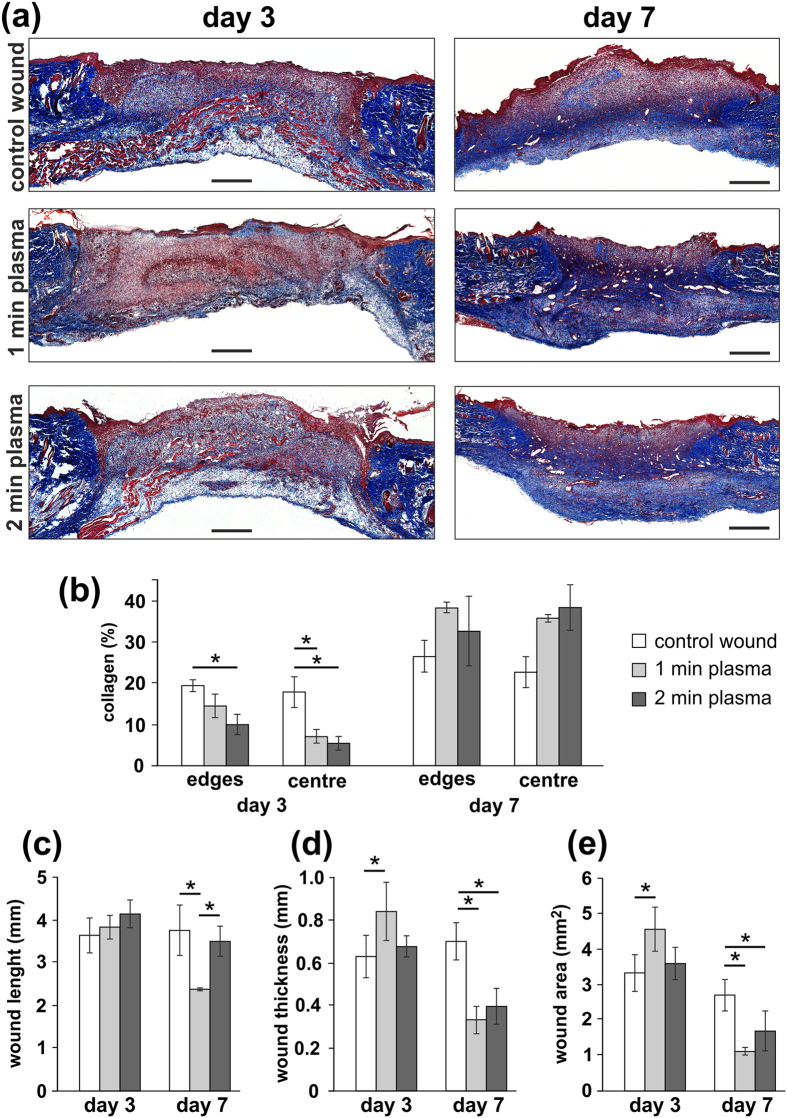
(**a**) Representative histology of Masson’s trichrome staining of control and plasma treated wounds on days 3 and 7. Scale bars: 500 μm. (**b**) Quantitative analysis of collagen staining (blue) density within the scar. Collagen staining intensity in the plasma treated group was significantly higher than that in the control on day 3, but not on day 7 after the wounding. (**c**–**e**) Quantitative analysis of (**c**) length, (**d**) thickness and (**e**) wound area in control and plasma treated wounds on days 3 and 7 after the wounding. Significantly lower thickness and wound area were found after plasma treatment on day 7. Mean ± SEM, *p < 0.05, n = 5.

Using Masson’s trichrome staining, the distribution of collagen within the wound area and the morphological parameters, such as the length of the linear epithelial gap, thickness, and the area of the granulation tissue were assessed using quantitative image analysis (Fig. 4). As is apparent on Fig. 4a,b, the collagen deposits were reduced in the wound area, while plasma treatment significantly reduced new collagen formation within the granulation tissue of the wound on day 3 (p < 0.05). Collagen formation was then enhanced on day 7, while a higher (but not significant) collagen deposition was found in plasma treated groups than in control groups (Fig. 4b).

**Figure 5:**
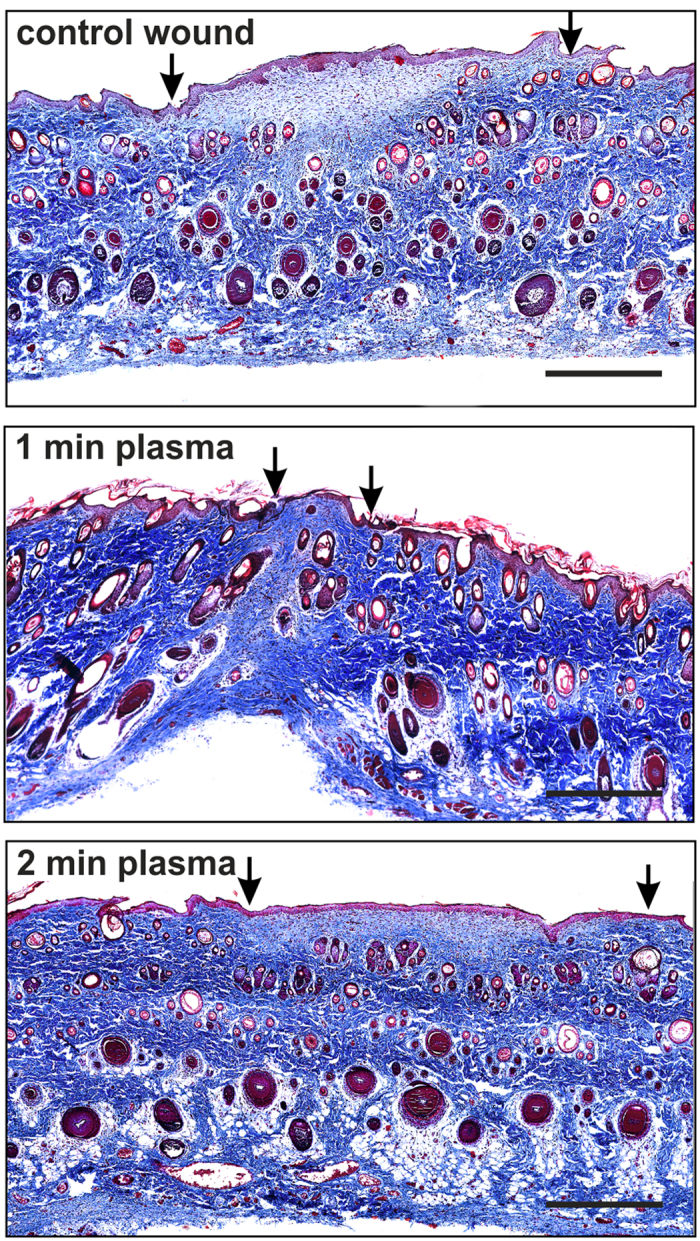
Representative histology of Masson’s trichrome staining of control and plasma treated wounds on day 14. The wounds were completely epithelised. The arrows indicate the edges of the remaining scar. Scale bars: 500 μm.

Correspondingly, the length of the epithelial gap did not differ between experimental groups on day 3, while a significantly shorter epithelial gap was found after 1 min of plasma treatment when compared to the control wounds, and wounds treated with plasma for 2 min on day 7 (Fig. 4c, p < 0.05). The thickness of the granulation tissue, as well as the area of the granulation tissue reduced significantly after plasma treatment on day 7 (p < 0.05), suggesting a positive effect of the plasma treatment on wound contraction, and re-epithelisation in the later phase of the healing (Fig. 4d,e). Figure 5 then shows full epithelialisation and collagen deposition in the wound area on day 14, when the wounds have fully closed, and the wound area is replaced by a completely developed skin structure, apart from a small area of residual scar tissue.

**Figure 6:**
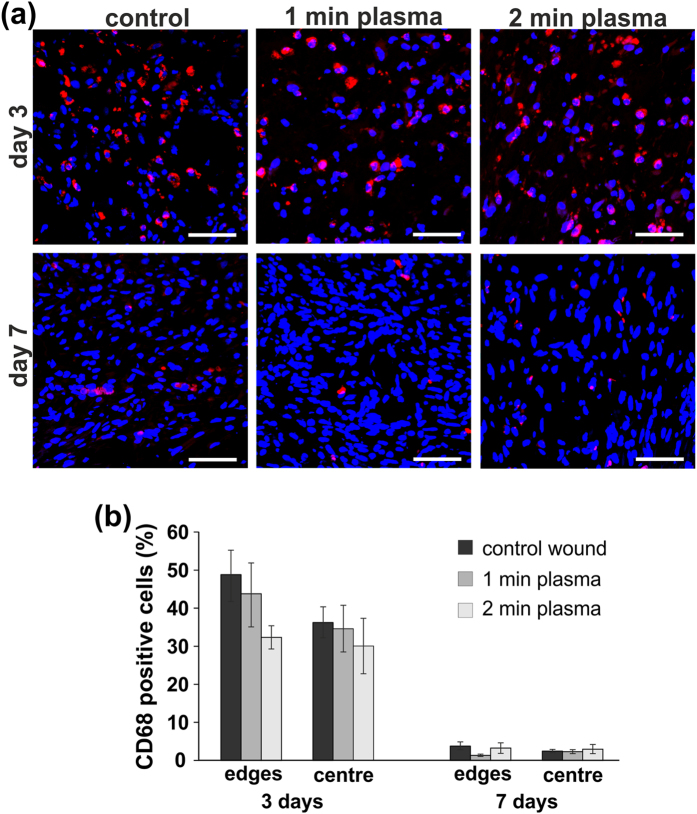
(**a**) Representative micrographs of CD68 immunostaining for macrophages (red) and cell nuclei (blue) in control and plasma treated wounds, 3 and 7 days after wounding. (**b**) Quantification of CD68 positive cells. The number of CD68 positive cells did not significantly differ in control and plasma treated wounds (wound edges and wound centre). Mean ± SEM, n = 5. Scale bars: 50 μm.

Furthermore, analysis of CD68 immunofluorescent staining on days 3 and 7 did not show any significant differences in the macrophage infiltration in the wound site after plasma treatment, compared to the control wounds (Fig. 6). The number of CD68 positive cells rapidly decreased on day 7, when only a few CD68 positive cells were detected in both control and plasma treated wounds.

### Analysis of mRNA Expression

**Figure 7:**
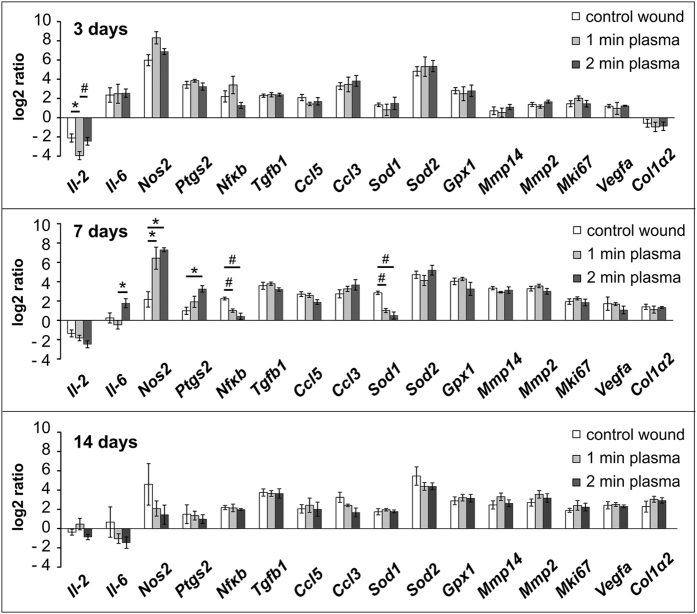
Gene expression profiling in the control and plasma treated wounds 3, 7 and 14 days after wounding. The graphs show the log2-fold changes of the ΔΔCt values of the indicated genes in comparison to the unwounded skin values. Mean ± SEM, *p < 0.05, ^#^p < 0.005, n = 5.

Changes in the mRNA expression of selected genes were determined at 3, 7 or 14 days after wound lesioning, and compared to the expression levels in the intact unwounded skin (Fig. 7, Table 1). From all the observed genes, the significant changes were found in expressions of *Il-6, Ptgs2, Nos2, Sod1* and *NF_K_b* on day 7, while the other observed genes did not significantly differ after plasma treatment at any time intervals in comparison to the control wounds.

Importantly, no significant changes between control and plasma treated wounds were detected on day 3, except of a more profound decrease in expression of *Il-2* after 1 min of plasma treatment (p < 0.05). On day 7, significant upregulation in expression of *Nos2* was found after 1, as well as 2 min of plasma treatment (p < 0.05), while upregulation of *Ptgs2* was found after only 2 min of plasma treatment (p < 0.05). Conversely, expressions of *NF_K_b* and *Sod1* significantly decreased after both 1 and 2 min of plasma treatment when compared to the control wounds (p < 0.005). Inflammatory markers *Il-6, Ptgs2* as well as *Nos2* declined on day 14, while upregulation persisted for the other observed genes, and no significant changes were found between control and plasma treated wounds.

## Discussion

Wound healing comprises a cascade of multiple biological and biochemical processes, which consist of a series of phases involving blood clotting, inflammation, cell proliferation, migration and differentiation, leading to the formation of granulation tissue, wound contraction, and final tissue restoration and remodelling. The key roles in these processes are played by growth factors, cytokines and inflammatory mediators released at the wound site^29^. Alterations that disrupt controlled healing processes may then lead to chronic or non-healing wounds, or excessive fibrosis.

Chronic non-healing wounds with a persistence of various bacteria represent an increasing health problem worldwide associated with high mortality, morbidity, and economic expenses. This emphasises the need for the development of new concepts and strategies for the elimination of bacterial load, and an improvement of chronic wound care. Based on its non-specific antimicrobial effects, NTPs appear to be a promising biomedical tool for effectively eliminating wound contamination and restarting the normal wound healing process. Indeed, several clinical trials have already approved the potential of NTP in the elimination of bacterial load, as well as for improved chronic wound healing^20^,^21^,^22^.

In the present study, we evaluated the efficacy of our NTP system in the healing of acute skin wounds in rat models. While most of the already published studies on wound healing were carried out using argon plasma^16^,^17^,^18^, we applied air plasma, which generates high amounts of reactive nitrogen species, such as nitric oxide (NO), nitrogen dioxide (NO_2_) and reactive oxygen species such as ozone (O_3_), superoxide (O_2_^−^) and hydroxyl radicals (∙OH). In contrast to air plasma, the composition of the argon plasma significantly differs, and only low amounts of reactive oxygen and nitrogen species are produced, by mixing the argon plasma with the ambient air.

The distinct effects of argon and air plasma are apparent from the experiments *in vitro* on cell cultures. While several reports demonstrated that argon NTP promotes cell migration and proliferation, NTP generated from air induced cell death, due to the apoptosis or necroptosis related mechanisms^26^,^27^. Despite the dramatically different effect *in vitro*, the enhancing plasma effect on wound healing was demonstrated in both argon^16^,^17^,^18^ and air plasma^22^. Of note, the comparability of these particular studies is disputable, as there is remarkable variability between the plasma generators, plasma dose and voltage value producing the plasma discharges.

Initially, we observed wound contraction, which is a primary healing mechanism in rodents. The wound area was significantly more reduced in the rats treated with NTP than in that of the untreated rats on day 7, and also histological analysis confirmed an enhanced epithelisation and a reduced granulation tissue area after plasma treatment. Respectively, improved wound contraction after the plasma treatment has also been confirmed in other studies using NTP generated from argon, helium or nitrogen1^6^,^7^,^8^,^9^,^10^,^11^,^12^,^13^,^14^,^15^,^16^,^17^,^18^,^30^.

According to histological evaluation, reduced collagen deposition in the wound area in the early inflammatory phase of healing was lower after plasma treatment, than that in the control group. This might indicate that plasma treatment slows down the collagen remodelling in the early phase of the healing process. However, in accordance to the wound healing progression and tissue remodelling in the later phases of the healing, collagen deposition gradually increased in all groups without any significant differences, and the skin layers were nearly fully remodelled on day 14 without any signs of abnormal scarring.

The inflammatory reaction, which was investigated by analysis of macrophage infiltration in the wound area, was not affected by plasma treatment. Similarly, mRNA analysis revealed an upregulation of inflammatory markers *Il-6, Nos2* and *Ptgs2* during the inflammatory phase of the healing (day 3) in both control as well as plasma treated wounds, without significant differences. However, while the level of expression of these factors declined in control wounds on day 7, an upregulation of *Nos2, Il-6* and *Ptgs2* persisted after 2 min of plasma treatment.

Our results are in line with the findings of the study by Arndt *et al*., who used daily, a 2 min argon NTP treatment of skin wounds in mice, and found an increase expression of *Il-6,* and monocyte chemoattractant protein-1 five days after wounding^17^. Similarly, Yu *et al*. showed that argon plasma treatment promoted acute inflammation^18^.

In particular, inducible nitric oxide synthase (NOS2, iNOS) is an enzyme responsible for the endogenous NO release, which is a highly diffusible intercellular signaling molecule that has been shown to regulate many processes in human skin physiology. The beneficial effects of NO on wound repair may be attributed to its functional influences on angiogenesis, inflammation, cell proliferation, matrix deposition, and remodelling^31^. Indeed, high iNOS levels were associated with higher healing rates in chronic leg ulcers^32^, while impaired wound repair was associated with lower NO bioavailability^33^.

As air plasma treatment is capable of increasing NO accumulation in the living tissue^16^,^34^, exogenic NO generated by the plasma may also stimulate NO synthesis through increased activity of iNOS. Enhanced NO concentration in the skin tissue might thus be the key molecular mechanism responsible for the plasma induced acceleration of the healing process, as well as wound closure. On the other hand, overexpression of iNOS may be attributed to excess collagen formations in keloid lesions^35^.

Notably, no significant changes between control and plasma treated wounds were found for the expression of genes related to wound remodelling, such as matrix metalloproteinase (*Mmp2* and *Mmp14*) or collagen (*Col1a2*), angiogenesis (*Vefga*), cell proliferation (*Mki67*), growth factors (*Egf, Fgf2, Pdgfa*) or macrophage polarization (*Cd86, Cd163*). This suggests that plasma exposure with the doses which are effective to kill bacteria has no harmful effects on a wound healing scenario nor will it promote excessive scar formation.

In conclusion, we have demonstrated that repeated 1 or 2 min air plasma treatment promoted healing of skin wounds in rats, by an improved wound closure in the proliferative phase of the healing. Of note, and similar to many other studies, we used a model of acute and sterile wound repair, which does not imply distinct pathophysiology of impaired healing in chronic and/or contaminated wounds. As NTP has bactericidal effects, its primary role in the wound healing process is to inhibit bacterial infection. In addition, in this study we showed that NTP can also stimulate wound healing by other indirect mechanisms. Overall, our data support a therapeutic use of air NTP for wound healing which is essential for the further development of NTP in clinics.

## Conclusions

In this study, we have demonstrated the safe and effective use of non-thermal atmospheric air plasma treatment for acute wound healing, highlighting the favourability of plasma applications for wound therapy. Among the main advantages of NTP treatment are the absence of noticeable side effects and the concomitant activation of pro-inflammatory signalling. A possibility to combine the biological and physical effects triggered by NTP opens a wide range of clinical applications for this technique.

## Materials and methods

### Physicochemical characterization of the plasma

To produce uniform non-thermal plasma for biological applications, we utilised a single jet NTP system, generating atmospheric pressure air plasma with ion volume density 5 · 10^17^ m^−3^, and gas temperature 30–35 °C. During the treatment time Δt = 1 min, the plasma produced a mean ion fluency of 6.4 · 10^14^ ions cm^−2^. Detailed characteristics of the system have been published previously^10^,^27^,^28^.

The antibacterial efficacy of air NTP was demonstrated on the Gram-negative (Pseudomonas aeruginosa), and the Gram-positive bacteria (Staphylococcus aureus). The bactericidal efficacy depended on the treatment time, and reached a 99.999% reduction of Gram-positive and a 99.9999% reduction of Gram-negative bacteria after 60 s of plasma exposure (Supporting information Fig. 1).

### Wound healing model

All experiments were performed in accordance with the European Communities Council Directive of 22nd September 2010 (2010/63/EU) regarding the use of animals in research and were approved by the Ethics Committee of the Institute of Experimental Medicine, Academy of Sciences of the Czech Republic, in Prague.

Forty one male Wistar rats (18 control animals, 23 plasma treated animals) with body weight in the range of 280–320 g were used in the study.

One day before wounding, the animals were placed under isoflurane anesthesia (2%, Forane, Abbott Laboratories, Abbott Park, IL), and the hair on their backs was removed using an electric shaver and depilatory cream (Weet, Reckitt Benckiser, Czech Republic). The following day, two full-thickness excisions with 8 mm in diameter were created by punch biopsy (Stiefel Laboratories Ltd., Sligo, Ireland) on the rats’ upper back midline, under sterile conditions. The plasma treated wounds were exposed for 1 min (right side) and 2 min (left side) to NTP with a distance of 1 cm from the plasma jet. To reduce pain, caprofen (4.4 mg/kg, Rimadyl, Zoetis, Parsippany, NJ) was injected subcutaneously near the wound area immediately after wounding.

The wounds were then covered with Vaseline Gauzes dressing (Steriwund Ltd., Czech Republic), gauze Mesalt (Mőlnlycke Health Care AB, Gothenburg, Sweden) and fixed with Tegaderm™ (3 M, St. Paul, MN, USA) and a self-adhesive elastic bandage (Idealast-haft, Paul Hartmann AG, Germany), which was enfolded around the animal body, with trimmed holes for the forelimbs. The wounds were exposed to additional plasma treatments on days 1 and 2 after wound generation, with a total of three daily plasma treatments during the experiment. The control animals underwent the same procedure as plasma treated animals, only without exposure of the wound to plasma. The animals were euthanized on days 3, 7 and 14 after the wounding, and skin samples including the wound region were harvested for further assessments.

### Wound healing analysis

Digital photographs of the wounds were taken using a digital camera (Canon PowerShot A650IS, Tokyo, Japan) at days 0–3, while n_c_ (control wound) = 18 and n_p_ (plasma treated wound) = 23, on days 7 (n_c_ = 14, n_p_ = 18), and on days 10 and 14 (n_c_ = 7, n_p_ = 8). The surface area of the wound was determined by tracking the wound margins and calculating the wound area using ImageJ software (NIH, Bethesda, MD). The percentage of wound area was calculated as (wound area of measured wound at a given time interval/area of original wound on day 0) ×100.

### Histological staining and analysis

The skin samples were fixed in 4% paraformaldehyde (in 0.1 M PBS, pH 7.4) and mounted into paraffin. A series of 5 μm thick paraffin sections were stained with hematoxylin/eosin (H&E) and Masson’s trichrome stain (Sigma-Aldrich, Steinheim, Germany) for histological analysis. To identify macrophages, a mouse monoclonal antibody ED-1directed against CD68 (1:150, Abcam, Cambridge, UK) and AlexaFluor 594-conjugated goat anti-mouse IgG (1:400; Life Technologies, Eugene, OR, USA) secondary antibody were used. The nuclei were visualised using 40, 6-diamidino-2-phenylindole (DAPI) fluorescent dye (Invitrogen, Paisley, UK). Images were acquired by a laser scanning confocal microscope (LSM 510 DUO, Zeiss, Jena, Germany) and Leica microscope (Olympus Optical, Hamburg, Germany).

Quantitative image analysis for collagen deposition was performed on sections stained with Masson’s trichrome stain. Three randomly selected sections from each animal (n = 5) were scanned on the Leica microscope using a 20x objective and used for the analysis. Three photos from each slide were taken in the centre, and the edges of the wound and the relative area of collagen was determined using HistoQuest Analysing Software (TissueGnostics GmbH, Vienna, Austria).

Skin samples stained with Masson’s trichrome were also used for histological assessment of the wound remodelling. The extent of epithelialisation was determined by measuring the linear epithelial gap, characterised as the length of a line segment between the margins of the ulcer. The thickness of the granulation tissue was measured at the wound centre. The wound area was determined as an area of the granulation tissue between the margins of the ulcer.

The number of macrophages was quantified as a fraction (%) of CD68 positive cells in relation to the total cell number of cells determined by DAPI staining in the analysed area. Three randomly selected sections from each animal (n = 5) were scanned on the Leica microscope using a 20x objective and used for the analysis. Three photos from each slide were taken in the centre, and the edges of the wound and analysed using HistoQuest Analysing Software (TissueGnostics GmbH, Vienna, Austria).

### Analysis of mRNA expression by quantitative real-time PCR (qPCR)

Changes in the mRNA expression of genes related to inflammation (*Il-2, Il-6, Il-10, Nos2, Ptgs2, *NF_k_b*, Tgfb1, Ccl3, Ccl5, Cd86, Cd163*), oxidative stress (*Sod1, Sod2, Gpx1*), matrix metalloproteinases (*Mmp2, Mmp14*), tissue remodelling (*Col1a2*), growth factors (*Egf, Fgf2, Ctgf* ), angiogenesis (*Vegfa*), proliferation (*Mki67*), and apoptosis (*Bax*) were studied using quantitative real-time reverse transcription polymerase chain reaction (qPCR) at 3, 7 or 14 days after the wounding (in all groups n = 5). RNA was isolated from tissue sections using the RNeasy Fibrous Tissue Mini Kit (Qiagen, Hilden, Germany), following the manufacturer’s recommendations. RNA amounts were quantified using a spectrophotometer (NanoPhotometer™ P-Class, Munchen, Germany). The isolated RNA was reverse transcribed into cDNA using Transcriptor Universal cDNA Master (Roche) and a thermal cycler (T100™ Thermal Cycler, Bio-Rad, Hercules, CA, USA). The qPCR reactions were performed using cDNA solution, FastStart Universal Probe Master (Roche, Germany) and TagMan^®^ Gene Expression Assays (Supplementary information Table 1, Life Technologies, Carlsbad, CA, USA).

The qPCR was carried out in a final volume of 10 μL containing 75 ng of extracted RNA. Amplification was performed on the real-time PCR cycler (StepOnePlus™, Life Technologies). All amplifications were run under the same cycling conditions: 2 min at 50 °C, 10 min at 95 °C, followed by 40 cycles of 15 s at 95 °C and 1 min at 60 °C. All samples were run in duplicate, and a negative control was included in each array. Relative quantification of gene expression was determined using the ΔΔCt method. Results were analyzed with StepOnePlus^®^ software. The gene expression level was normalized on *Gapdh* as a reference gene; control samples from the intact animals were used as a calibrator.

### Statistical analysis

All data were expressed as mean ± standard error mean (SEM). The statistical significance of differences between the treated groups and the control group was analyzed using one-way ANOVA with Holm-Sidak multiple parameter test (Sigmastat 3.1, Systat Software Inc., San Jose, CA, USA) with a level of p < 0.05 considered statistically significant.

## Additional Information

**How to cite this article:** Kubinova, S. *et al*. Non-thermal air plasma promotes the healing of acute skin wounds in rats. *Sci. Rep.* **7**, 45183; doi: 10.1038/srep45183 (2017).

**Publisher's note:** Springer Nature remains neutral with regard to jurisdictional claims in published maps and institutional affiliations.

## Supplementary Material

Supplementary Information

## Figures and Tables

**Table 1:** mRNA gene expression of selected genes.

	day 3			day 7		
	control	1 min plasma	2 min plasma	control	1 min plasma	2 min plasma
*Il10*	0.76 ± 0.72	0.57 ± 0.35	0.65 ± 0.45	0.41 ± 0.45	−0.28 ± 0.24	−0.40 ± 0.42
*Egf*	−2.41 ± 0.38	−2.31 ± 0.23	−2.28 ± 0.34	−2.22 ± 0.22	−1.92 ± 0.22	−2.53 ± 0.14
*Fgf2*	0.71 ± 0.21	0.60 ± 0.26	0.56 ± 0.10	0.93 ± 0.84	0.58 ± 0.32	−0.02 ± 0.12
*Ctgf*	0.63 ± 0.18	0.80 ± 0.43	0.50 ± 0.22	1.04 ± 0.43	1.07 ± 0.24	0.72 ± 0.27
*Cd86*	1.15 ± 0.14	0.80 ± 0.43	1.13 ± 0.36	2.06 ± 0.18	−2.24 ± 0.33	1.74 ± 0.08
*Cd163*	3.71 ± 0.20	3.74 ± 0.55	3.95 ± 0.57	2.18 ± 0.34	1.62 ± 0.26	1.76 ± 0.40
*Bax*	0.46 ± 0.09	0.64 ± 0.46	0.34 ± 0.22	1.67 ± 0.22	2.08 ± 0.33	1.54 ± 0.16

The values are expressed as the log2-fold changes of the ΔΔCt values of the indicated genes in comparison to the unwounded skin values. Mean ± SEM, n = 5.
