# Supplementary material for: Non-thermal air plasma promotes the healing of acute skin wounds in rats
Source: Sci Rep. 2017 Mar 24;7:45183. doi: 10.1038/srep45183 (PMC5364525; doi:10.1038/srep45183)
Supplement: Supplementary Information [file srep45183-s1.docx]

**Supplementary information:**

**Non-thermal air plasma promotes the healing of acute skin wounds in rats**

S Kubinova^1,2^, K Zaviskova^1,3^, L Uherkova^1^, V Zablotskii^2^, O Churpita^2^, O Lunov^2^, A Dejneka^2^

^1^Institute of Experimental Medicine, Academy of Sciences of the Czech Republic, Prague, Czech Republic

^2^Institute of Physics, Academy of Sciences of the Czech Republic, Prague, Czech Republic

*^3^2^nd^ Medical Faculty, Charles University, Prague, Czech Republic*

Corresponding author:

Sarka Kubinova, PhD

Institute of experimental medicine AS CR, Videnska 1083, 1420 Prague, Czech Republic

E-mail: [sarka.k@biomed.cas.cz](mailto:sarka.k@biomed.cas.cz)

Phone: +420 241 062 635

**Antibacterial effect of air plasma**

**Methods:**

To study the bactericidal effects of plasma we used the Gram-negative bacteria Pseudomonas aeruginosa (ATCC 27853), and the Gram-positive bacteria Staphylococcus aureus (ATCC 6538) (Czech Collection of Microorganisms (Brno, Czech Republic). The gelatine pellets containing the bacterial strains were incubated in 9 ml of liquid media (Tryptic Soy Broth, Mecrotube®, Merc, NJ, USA) at 35°C for 18 hours, and then diluted in a phosphate buffer (PBS) to a concentration 6 x 10^6^ colony forming units (CFU)/ml. A volume of 0.8 ml of diluted bacteria suspension was spread onto the agar plate (Caso-Agar, Mercoplate®, Merc) and exposed to plasma for 15, 30 and 60 s, with the distance 10 mm from the plasma jet. The plates were incubated overnight and the number of CFU in the inhibition zone was counted using ImageJ software (NIH, Bethesda, MD, USA). The tested layers were tested in triplicates. The bactericidal efficacy of the plasma treatment for P. aeruginosa and S. aureus was expressed as the log reduction [log (number of CFU before treatment) – log (number of CFU after the treatment)].

**Scanning electron microscopy (SEM)**

Changes in bacteria morphology after NTP irradiation were assessed by scanning electron microscope (SEM). P. aeruginosa and S. aureus were placed on glass coverslips and exposed to either plasma jet located 10 mm away, for 15, 30 and 60 s, followed by 4 % paraformaldehyde fixation. Afterwards, cells were post-fixed with 1% OsO_4_ at room temperature then dehydrated with a graded ethanol series followed by subsequent chemical drying with hexamethyldisilazane. Micrographs were taken using high resolution SEM FEI Quanta 3D FEG at an acceleration voltage of 2 kV to reveal surface details and prevent charging of the non-conductive samples.

**Results:**

Generally, it is well-known that aerobic and anaerobic microorganisms colonise the majority of dermal wounds ^1^. It has been observed that microbial density contributes to a delay in wound healing, progressing infection and resulting in chronicity of wounds ^1,2^. Moreover, antimicrobial resistance has emerged as a rapidly growing public health issue ^3^. Non-thermal plasma has shown great potential as an effective alternative approach in overcoming resistant microorganisms ^4,5^. Therefore, we firstly investigated how effective NTP will be in the eradication of bacteria. The antibacterial efficacy of air NTP is demonstrated on two types of bacteria (S. aureus and P. aeruginosa), which were found most frequently in different kinds of wounds ^1^.

The bactericidal efficacy depended on the treatment time, and reached a 99.999% reduction of both types of bacteria after 60s of plasma exposure (Supplementary Fig.1a). In addition, to study in detail morphological changes of bacteria after plasma treatment, we performed an SEM investigation of strains exposed to NTP. Within 15s of NTP treatment, both bacteria strains showed mild changes of morphological characteristics. Profound membrane damage was observed after 30s. After 60s of NTP exposure, cellular structures were clearly destroyed (Supplementary Fig.1b).


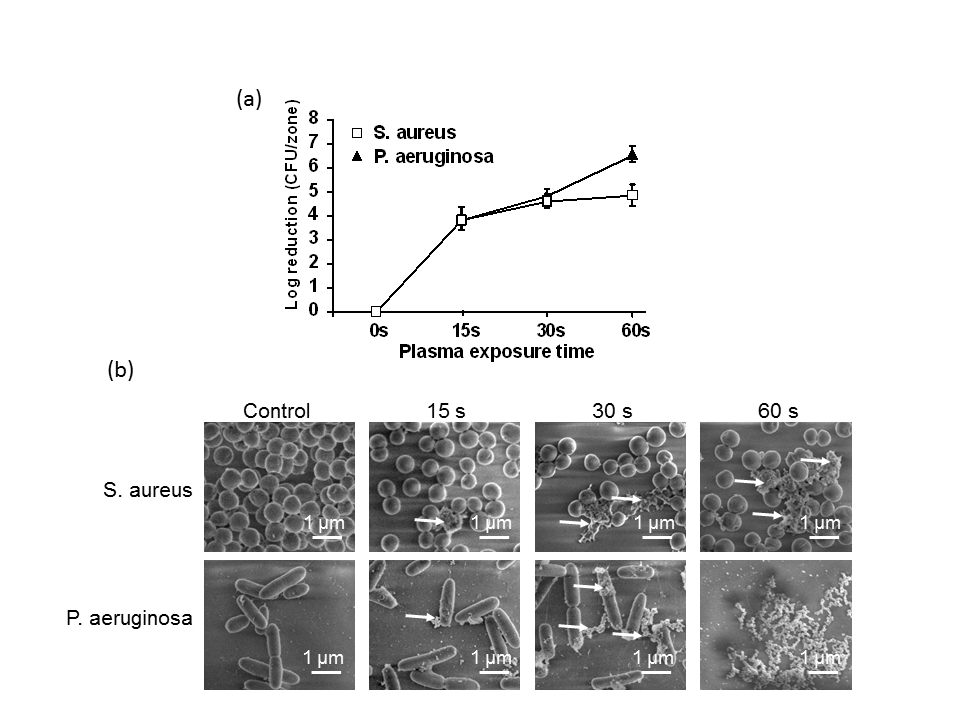


**Supplementary Figure 1.** (a) Bactericidal efficacy of NTP on Staphylococcus aureus and Pseudomonas aeruginosa, expressed as log reduction after 15, 30 and 60 s of NTP treatment. Mean ± SEM, n = 3. (b) Scanning electron micrographs of untreated (control) and NTP-treated bacterial strains.

**References:**

1 Bowler PG, Duerden BI, Armstrong DG. Wound microbiology and associated approaches to wound management. *Clin Microbiol Rev* 2001; **14**: 244-69.

2 Rahim K, Saleha S, Zhu X *et al.* Bacterial Contribution in Chronicity of Wounds. *Microb Ecol* 2016: 1–12.

3 Piddock LJV. The crisis of no new antibiotics-what is the way forward? *Lancet Infect Dis* 2012; **12**: 249-53.

4 Fridman G, Friedman G, Gutsol A *et al.* Applied plasma medicine. *Plasma Process Polym* 2008; **5**: 503-33.

5 Lunov O, Zablotskii V, Churpita O *et al.* The interplay between biological and physical scenarios of bacterial death induced by non-thermal plasma. *Biomaterials* 2016; **82**: 71-83.

Supplementary Table 1: TagMan® Gene Expression Assays

| gene |  | Catalog no. |
| --- | --- | --- |
| *Gapdh* | Glyceraldehyde 3-phosphate dehydrogenase | Rn01775763_g1 |
| *Il2* | Interleukin 2 | Rn00587673_m1 |
| *Il6* | Interleukin 6 | Rn01410330_m1 |
| *Il10* | Interleukin 10 | Rn 00563409_m1 |
| *Ccl3* | Chemokine (C-C motif) ligand 3 | Rn01464736_g1 |
| *Ccl5* | Chemokine (C-C motif) ligand 5 | Rn00579590_m1 |
| *Ptgs2* | Prostaglandin-endoperoxide synthase 2 | Rn01483828_m1 |
| *Nos2* | Nitric oxide synthase 2, inducible | Rn00561646_m1 |
| *Egf* | Epidermal growth factor | Rn00563336_m1 |
| *Ctgf* | Connective tissue growth factor | Rn01537279_g1 |
| *Cd86* | CD86 molecule | Rn00571654_m1 |
| *Cd163* | CD163 molecule | Rn01492519_m1 |
| *Mmp2* | Matrix metallopeptidase 2 | Rn01538170_m1 |
| *Mmp14* | Matrix metallopeptidase 14 (membrane-inserted) | Rn00579172_m1 |
| *Col1a2* | Collagen, type I, alpha 2 | Rn01526721_m1 |
| *Vegfa* | Vascular endothelial growth factor A | Rn01511601_m1 |
| *Fgf2* | Basic fibroblast growth factor | Rn00570809_m1 |
| *Mki67* | Marker of proliferation Ki-67 | Rn01451446_m1 |
| *Bax* | Bcl2-associated X protein | Rn02532082_g1 |
| *Tgfb1* | Transforming growth factor, beta 1 | Rn00572010_m1 |
| *Pdgfa* | Platelet-derived growth factor alpha polypeptide | Rn00709363_m1 |
| *Sod1* | Superoxide dismutase 1, soluble | Rn00566938_m1 |
| *Sod2* | Superoxide dismutase 2, mitochondrial | Rn00690588_g1 |
| *Gpx1* | Glutathione peroxidase 1 | Rn00577994_g1 |
| *Nfkb1* | Nuclear factor of kappa light polypeptide gene enhancer in B-cells 1 | Rn01399572_m1 |
